# MET receptor is a potential therapeutic target in high grade cervical cancer

**DOI:** 10.18632/oncotarget.3161

**Published:** 2015-04-04

**Authors:** Katarzyna Miekus, Marta Pawlowska, Małgorzata Sekuła, Grazyna Drabik, Zbigniew Madeja, Dariusz Adamek, Marcin Majka

**Affiliations:** 1 Department of General Biochemistry, Faculty of Biochemistry, Biophysics and Biotechnology, Jagiellonian University, Cracow, Poland; 2 Department of Transplantation, Polish-American Institute of Pediatrics, Jagiellonian University Medical College, Cracow, Poland; 3 Department of Clinical Immunology, Polish-American Institute of Pediatrics, Jagiellonian University Medical College, Cracow, Poland; 4 Department of Cell Biology, Faculty of Biochemistry, Biophysics and Biotechnology, Jagiellonian University, Cracow, Poland; 5 Department of Pathomorphology, Jagiellonian University Medical College, Cracow, Poland

**Keywords:** cervical cancer, MET, CXCR4, differentiation, E-cadherin

## Abstract

Cervical cancer is one of the leading causes of death among women suffering from tumors. Current treatment options are insufficient. Here, we investigated the MET receptor as a potential molecular target in advanced cervical cancer. Downregulation of MET receptor expression via RNA interference in different cervical carcinoma cell lines dramatically decreased tumor growth and forced tumor differentiation *in vivo*. MET receptor silencing also led to a dramatic decrease in cell size and a decrease in proliferation rate under normal and stress conditions. MET receptor downregulation also resulted in decreased cyclin D1 and c-myc levels but did not increase apoptosis. Subsequent experiments showed that downregulation of the MET receptor decreased the expression of a key regulator of the epithelial-to-mesenchymal transition, SLUG. and increased the expression of E-cadherin, a hallmark of the epithelial phenotype. Moreover, MET downregulation impairs expression and signaling of CXCR4 receptor, responsible for invasive phenotype.

Taken together, our results strongly suggest that the MET receptor influences the oncogenic properties of cervical carcinoma cells *in vitro* and *in vivo*. These findings highlight a unique role of the MET receptor in cervical carcinoma cells and indicate the MET receptor as a potential therapeutic target for advanced cervical carcinoma.

## INTRODUCTION

Despite the recent development of a vaccine against human papillomavirus infection, cervical cancer (CC) remains the third leading cause of death among women suffering from cancer, especially in low- and middle-income countries [1, 2]. The survival rate for CC is related to the stage of the disease with distant metastasis. There is currently no effective therapy for advanced cervical cancer, and it is thus important to identify molecular markers that can help in the development of new therapeutic strategies to improve cervical cancer treatment.

The MET receptor belongs to the family of growth factor receptors with intrinsic tyrosine kinase activity and, together with its ligand, Hepatocyte Growth Factor (HGF), is involved in proliferation, inhibition of apoptosis, motility and dissemination of cancer cells during tumor development and progression [3, 4]. HGF and the MET receptor are also known to mediate epithelial-to-mesenchymal transitions (EMT) during embryonic development [5, 6] and tumorigenesis [7, 8].

EMT is a stage of metastasis in which epithelial cells acquire characteristics of mesenchymal stem cells [9]. EMT is regulated by a number of transcription factors, including SNAIL1, SLUG, ZEB1, ZEB2, and TWIST [10, 11] and is characterized by E-cadherin suppression, increased levels of N-cadherin and vimentin expression, and a highly invasive mesenchymal phenotype [12, 13, 14]. The MET receptor is overexpressed in a variety of human cancers, including thyroid carcinoma [15], ovarian cancer [16], colorectal cancer [17], rhabdomyosarcoma [18], and renal cell carcinoma [19]. It has been also shown that hepatocyte growth factor (HGF) and its receptor MET are significantly increased in ovarian cancer patients who relapse shortly after chemotherapy [20]. In cervical carcinoma, the MET receptor has been shown to promote scattering and morphogenesis of tumor cells [20, 21]. We previously showed that stimulation of the MET receptor with HGF alone or together with stromal-derived factor-1 (SDF-1), the ligand for the CXCR4 receptor, results in migration and cytoskeletal changes in cervical carcinoma cells [22]. Our data also showed that activation of both MET and CXCR4 caused the translocation of β-catenin into the nucleus and the subsequent upregulation of cyclin D1 expression [22].

In the present study, we used cell lines as well as tumor tissues to evaluate the role of the MET receptor in cervical cancer. We investigated whether the MET receptor is involved in the growth and progression of human cervical carcinoma *in vitro* and *in vivo* and whether silencing of this gene can change the cellular phenotype into a more benign form and thereby arrest cancer development. We demonstrate, for the first time, that downregulation of the MET receptor can reverse the EMT-driven cervical cancer cell phenotype into a more epithelial and less aggressive form. We further show that the MET receptor might be a promising target for advanced cervical cancer therapy.

## RESULTS

### MET receptor expression in patients' samples

**Figure 1:**
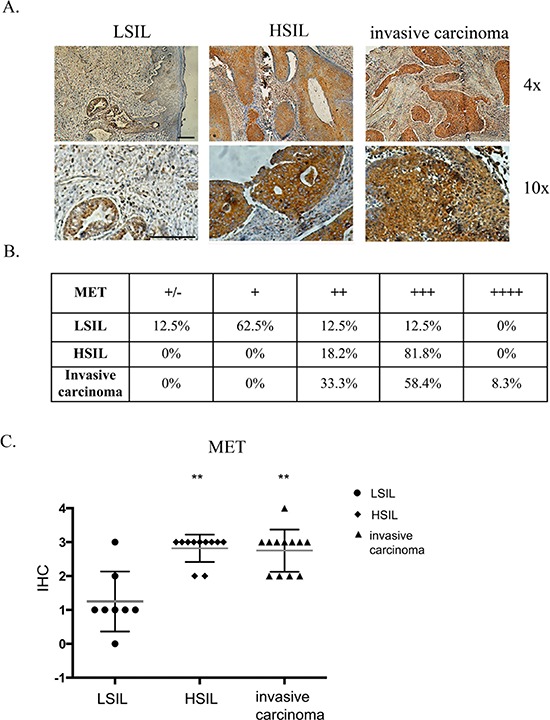
Immunohistochemical analysis of MET receptor expression in patient samples **(A)** Examples of immunohistochemical staining of MET receptor for LSIL, HSIL and invasive carcinoma. **(B** and **C)** Immunohistochemical analysis of MET receptor expression in human samples. In order to perform expression analysis we used the following scale: 0 (+/−) – very poor response/poorly positive discontinuous, 1 (+) – poor response, 2 (++) – moderate response, 3 (+++) – quite strong/strong response, 4 (++++) – very strong response. Samples were obtained from patients with mild, moderate or severe dysplasia and invasive cervical carcinoma. LSIL – Low-grade squamous Intraepithelial Lesion, HSIL – High-grade squamous Intraepithelial Lesion (according to Bethesda system terminology). The evaluation of the samples was performed with the approval from the Bioethics Committee of the Jagiellonian University (no. KBET/7/B/2008). *n* = 31, **p < 0.001. Bar = 200 μm.

Immunohistochemical analysis of 31 patients' tissues revealed that MET receptor expression varies depending on the grade of the tumor (Figure 1). Examples of immunohistochemical staining of LSIL, HSIL and invasive carcinoma are presented in Figure 1A. In order to perform staining analysis of MET receptor we used the scale from 0 to 4, where 0 (+/−) – very poor response/poorly positive discontinuous, 1 (+) – poor response, 2 (++) – moderate response, 3 (+++) – quite strong/strong response, 4 (++++) – very strong response. The immunohistochemical analysis revealed strong positive staining for MET receptor in over 80% of HSIL samples and strong and very strong positive reaction for 67% of invasive carcinoma (Figure 1B). Histopathological examination also showed that LSIL was characterized mainly by a poor expression of MET receptor (+). Strong (+++) and very strong (++++) MET expression we observed for samples described as HSIL and invasive carcinoma (Figure 1C).

### MET downregulation reduces the viability/proliferation of MET-deficient cells under stress conditions

**Figure 2:**
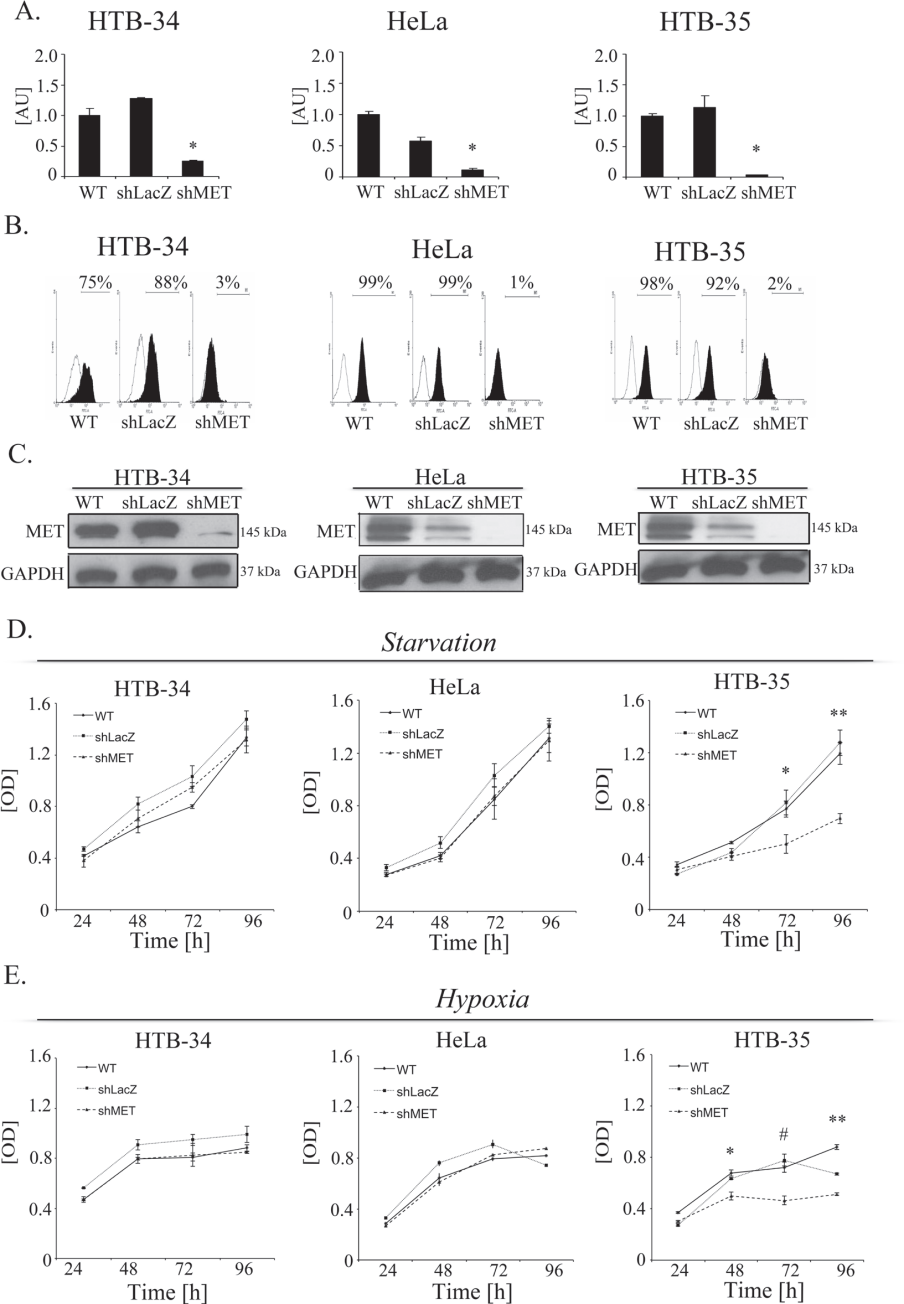
MET downregulation alters proliferation/viability under stress conditions MET receptor downregulation via a lentiviral vector containing anti-MET shRNA resulted in decreases at mRNA **(A)** and protein **(B, C)** levels. Downregulation of MET receptor alters proliferation/viability under hypoxia **(D)** and starvation **(E)** conditions. A – Real-time RT-PCR revealed significant decreases in MET transcript levels in MET receptor-silenced cells (shMET) relative to controls (wild type, WT, and shLacZ) in all tested cell lines (HTB-34, HeLa and HTB-35). B – Flow cytometry analysis of MET receptor expression. C – Western blot analysis revealed complete downregulation of MET receptor expression in shMET HTB-34, HeLa and HTB-35 cells. D. MTT assay of cells cultured under starvation conditions (MEM supplemented with 0.5% BSA). E – MTT assay of cells cultured under hypoxic conditions (2% oxygen). Western blot and FACS analyses were performed at least three times with similar results; representative results are shown. Real-time RT-PCR was performed at least three times in duplicates. MTT assay was repeated three times in triplicates. *p < 0.01, **p < 0.001.

Cervical carcinoma cells were transduced with lentiviral vectors containing anti-MET shRNAs that were established in our laboratory [23]. The efficiency of MET downregulation was assessed in cells transduced with control LacZ (shLacZ) and MET (shMET) shRNA and compared with control wild-type (WT) cells. MET receptor expression levels were evaluated at the mRNA level using real-time RT–PCR (Figure 2A) and at the protein level using flow cytometry (Figure 2B) and western blot analysis (Figure 2C). The functionality of the silenced receptor was tested by a chemotaxis assay (Supplementary Figure 1).

The growth of tumors *in vivo* induces specific conditions associated with limited access to oxygen and nutrients. The MET receptor promotes cell viability and proliferation during tumorigenesis [4]. To test whether the MET receptor influences cell viability/proliferation under stress conditions, cells were cultured in starvation medium (0.5% BSA) or under low oxygen (2%), and the MTT assay was performed. For the HTB-34 and HeLa cell lines, we did not observe any differences between control cells and MET-deficient cells under either starvation or low oxygen conditions (Figure 2D, 2E, left and middle panels). However, MET receptor downregulation significantly decreased the viability/proliferation of HTB-35 cells after 48 hours of starvation or hypoxic conditions. The largest difference between control and MET-deficient cells was reached after 96 hours of culture (Figure 2D, 2E, right panels). These data showed that MET receptor expression is important for viability/proliferation of HTB-35 cells under stress conditions. It has been already shown that some tumors are dependent on MET receptor activity for their growth and survival [24, 25]. In subsequent experiments we wanted to know whether MET receptor might be relevant for other characteristics of cervical cancer cells.

### MET receptor downregulation inhibits tumor growth *in vivo*

**Figure 3:**
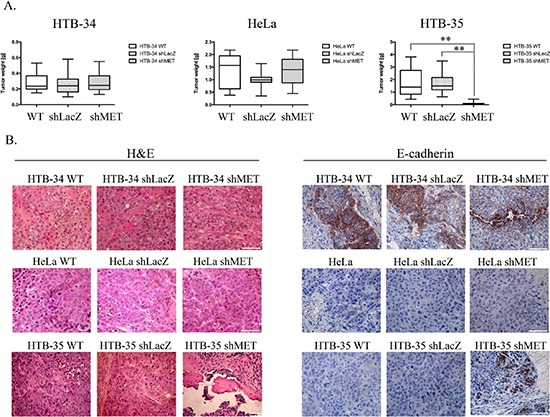
Tumor growth *in vivo* and histopathological analysis of control and shMET cervical carcinoma cells A comparison of tumors derived from control and shMET cervical carcinoma cells revealed differences in tumor weight, morphology and E-cadherin expression. **(A)** Tumor growth of WT, shLacZ and shMET cervical carcinoma cells was measured 30 days after subcutaneous injection of 5 × 10^6^ cells in NOD-SCID mice. **(B)** H&E staining showing that MET silencing did not influence the morphology of HTB-34 and HeLa tumors, whereas HTB-35 shMET tumors resembled more differentiated tumors with keratin pearls (arrows). E-cadherin is highly expressed in HTB-34 cells but absent in HeLa cells. Downregulation of MET in HTB-35 cells caused the re-expression of E-cadherin. These experiments were performed three times with *n* = 5; **p < 0.001. Representative staining is shown. Bar = 50 μm.

The reduced viability observed in the HTB-35 shMET cell line led us to study the influence of MET receptor silencing on cell behavior during tumor formation *in vivo*. To examine the growth of human cervical cell lines *in vivo,* we established a xenotransplant model in NOD-SCID mice. Mice were injected subcutaneously with 5 × 10^6^ WT, shLacZ or shMET cells. After 30 days, the mice were sacrificed, and the tumors were weighed. We observed that HTB-34 cells formed tumors with an average weight of 0.3 grams (Figure 3A, left panel), whereas tumors formed by HeLa cells weighed on average 1.3 grams (Figure 3A, middle panel). Despite this discrepancy in tumor weight, the growth of HTB-34 and HeLa tumors was not inhibited by MET receptor downregulation (Figure 3A, left and middle panels). Tumors generated by WT and shLacZ HTB-35 control cells did not differ in weight, forming tumors of approximately 1.7 grams (Figure 3A, right panel). Interestingly, shMET HTB-35 cells formed very small tumors, with a mean weight of only 0.08 grams (Figure 3A, right panel). Interestingly, 6 of 15 animals injected with HTB-35 shMET cells did not develop tumors at all.

A histopathological analysis of the tumor lesions revealed no differences between the tumors generated by control and MET-deficient HTB-34 and HeLa cells (Figure 3B, first and second panels). However, tumors formed by shMET HTB-35 cells clearly differed from their control counterparts. Here, our histopathological examination revealed that MET-deficient cells formed well-differentiated tumors with keratin pearls, i.e., whorl-shaped accumulations of keratin [26], whereas control tumors were poorly differentiated and composed of cells of various sizes and shapes with a large number of mitotic figures (Figure 3B, last panel). E-cadherin, a marker of the epithelial phenotype, was highly expressed in tumors generated by both control and shMET HTB-34 cells (Figure 3B, first panel). By contrast, both control and MET-deficient HeLa cells formed tumors that did not express E-cadherin (Figure 3B, second panel). Interestingly, MET receptor downregulation in HTB-35 cells led to the re-expression of E-cadherin *in vivo* (Figure 3B, last panel).

### Downregulation of the MET receptor inhibits cell proliferation by decreasing cyclin D1 and c-myc levels

**Figure 4:**
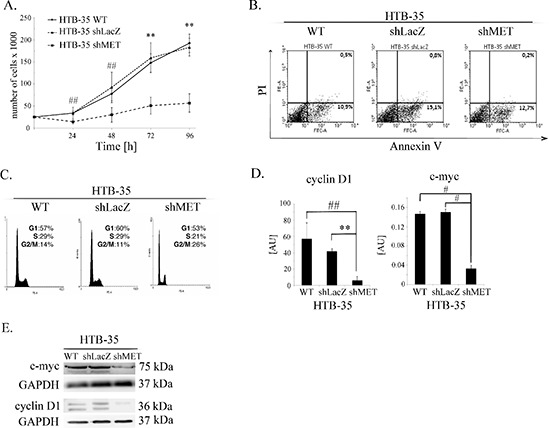
Influence of the MET receptor on cell function **(A)** Proliferation assay in HTB-35 cells. A significant decrease in proliferation rate was observed after MET silencing. Cells were cultured in medium supplemented with 10% FBS and counted every 24 hours. **(B)** Flow cytometry analysis of Annexin V and PI staining of control and MET-deficient HTB-35 cells did not reveal any differences. **(C)** Cell cycle analysis revealed that MET receptor downregulation decreased the proportion of cells in S phase and caused an accumulation in G2/M phase. **(D)** Real-time RT-PCR analysis of cyclin D1 and c-myc expression. Significant downregulation of cyclin D1 and c-myc was observed at the mRNA level after MET receptor downregulation. **(E)** Western blot analysis of c-myc and cyclin D1 protein expression. c-myc was weakly expressed in shMET HTB-35 cells, whereas cyclin D1 decreased to an undetectable level. Proliferation assay was performed three times in triplicate and real-time RT-PCR assays was performed three times in duplicate. FACS and western blot analyses were performed at least three times with similar results. Representative results are shown. **p < 0.001. ##. *p* < 0.005.

The inhibition of tumor growth observed in our *in vivo* studies directed our interest towards the properties of HTB-35 shMET cells. We first studied the growth of shMET cells. The cells were incubated under control conditions (10% FBS) and counted every 24 hours. We observed growth inhibition of shMET HTB-35 cells relative to WT and LacZ cells (Figure 4A). The difference in growth rate between control and shMET cells was significant at each time point tested. The proliferation rate of control cells was four times higher than that of shMET HTB-35 cells after 96 hours of culture.

To explore the reason for the decreased proliferation of MET-deficient cells, we analyzed apoptosis patterns and cell cycle progression. HTB-35 shMET cells did not undergo apoptosis spontaneously, and we did not observe any differences between control and shMET cells in early (AnnexinV^+^PI^−^) or late apoptosis (AnnexinV^+^PI^+^) (Figure 4B). However, there was a difference in cell cycle progression, as MET receptor downregulation decreased the number of cells in S phase and increased the number of cells in G2/M phase (Figure 4C). Cyclin D is known to regulate the entry into S-phase [27], and c-myc, as a transcriptional regulator, mediates processes such as proliferation, differentiation, transformation and prevention of apoptosis [28, 29]. Transcript levels of both genes were significantly downregulated after MET downregulation, with eight- and five-fold reductions observed for cyclin D1 and c-myc, respectively (Figure 4D). To confirm this result, we measured their protein levels in shMET cells by western blotting and detected only a faint signal for c-myc and no signal for cyclin D1 (Figure 4E). We also observed the influence of the MET receptor depletion on the expression level of genes correlated with invasive phenotype, angiogenesis and tumor growth. Downregulation of MET receptor decreases mRNA level of c-myc, CXCR4 and Slug and increases mRNA of HGF, CXCR7, HIF-1α, matrix metalloproteinases −2, −9 (MMP-2, −9) and tissue inhibitor of metalloproteinases −1, −2 (TIMP-1,−2). (Supplementary Table 1).

### MET downregulation alters cell morphology

**Figure 5:**
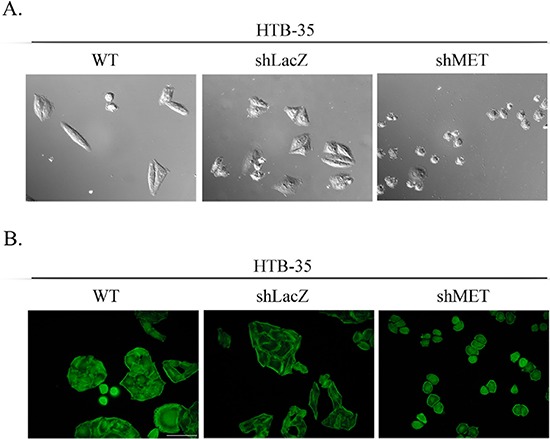
Cell morphology and actin organization after MET silencing An analysis of cell morphology revealed a decrease in cell size **(A)** and changes in F-actin organization **(B)** in MET-deficient HTB-35 cells. A – Cells were photographed with Nomarski interference contrast (upper panel) and analyzed with MIGRA software (lower panel). B – Immunofluorescence staining of the F-actin cytoskeleton shows re-organization of actin filaments from thick contractile bundles in control cells to thin cortical bundles – as in epithelial cells – in MET-deficient cells. Representative staining is shown. Bar = 50 μm., ## *p* < 0.005.

MET receptor downregulation significantly affected the morphology of HTB-35 cells. After transduction with the shMET lentiviral vector, the cells became small and rounded, whereas control cells resembled fibroblast-like cells with an elongated shape (Figure 5A). The values for all cell morphology parameters (area, periphery, extension, dispersion and elongation) were significantly lower in HTB-35 shMET relative to control cells. The data are summarized in Table 1. We also observed that MET downregulation altered F-actin organization. In MET-deficient HTB-35 cells, F-actin was located under the cell membrane and did not form regular stress fibers as observed in WT and shLacZ cells. Actin filaments reorganized from thick, parallel, contractile bundles in control cells to thin cortical bundles – as in epithelial cells – in MET-deficient cells (Figure 5B).

**Table 1:** The values for cell morphology parameters - area, periphery, extension, dispersion and elongation

Cell line	Area	Periphery	Extension^1^	Dispertion^1^	Elongation^1^
**HTB-35 WT**	885.85 ± 12.99	159.99 ± 5.87	1.05 ± 0.56	0.17 ± 0.32	0.88 ± 0.47
**HTB-35 shLacZ**	963.42 ± 423.58	178.63 ± 36.73	1.07 ± 0.51	0.16 ± 0.12	0.91 ± 0.46
**HTB-35 shMET**	343.94 ± 41.08^##^	90.62 ± 15.91^##^	0.24 ± 0.13^##^	0.03 ± 0.02^##^	0.22 ± 0.12^##^

1 Extension,dispersion and elongation take a value 0 if the shape is circular and increases without limit as the shape becomes less compact [51]. The mean value was obtained from analysis of 50 cells. Statistically significant at ## *p* < 0.005.

### E-cadherin and slug expression in patients' samples

**Figure 6:**
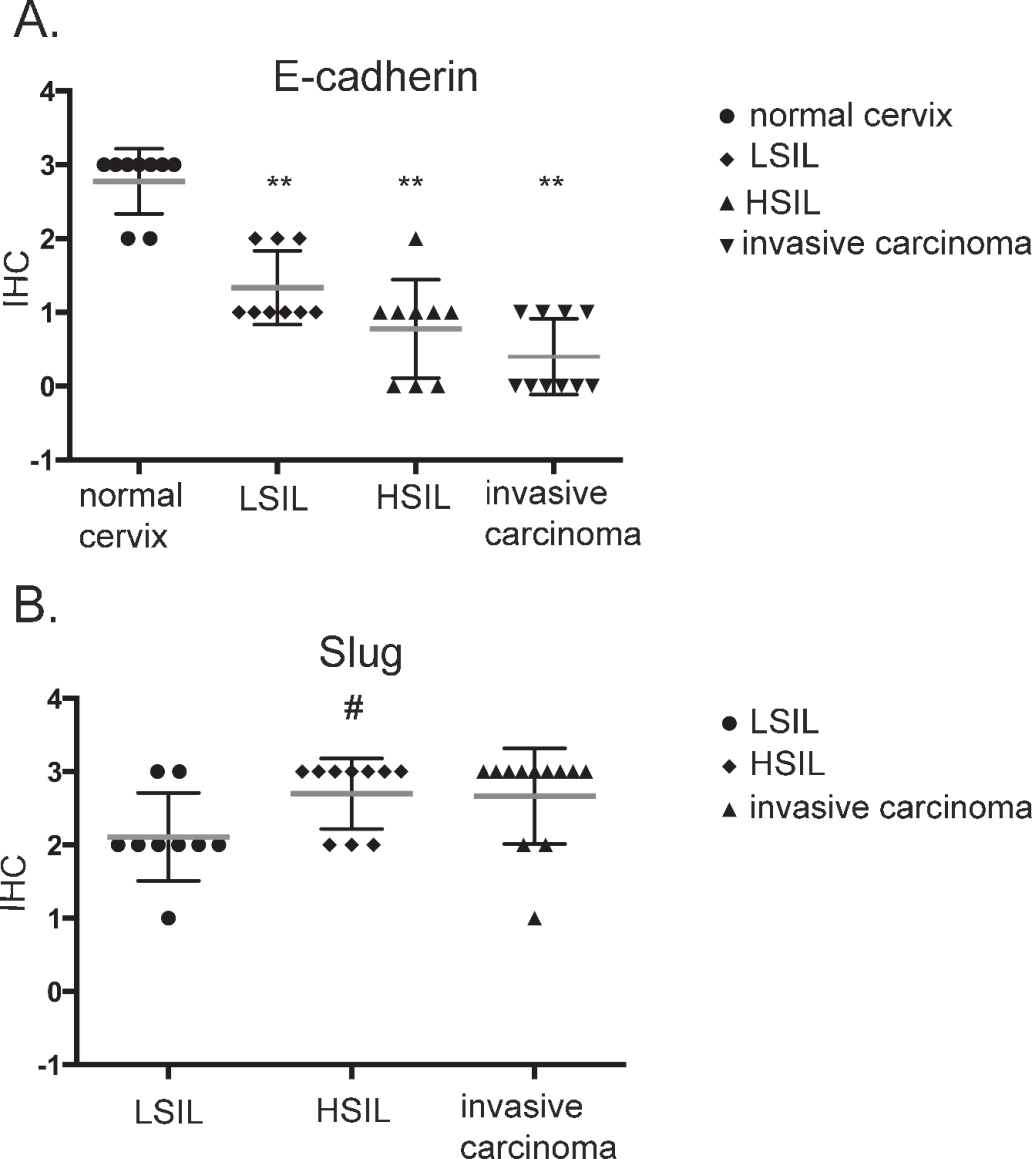
Immunohistochemical analysis of E-cadherin (A) and Slug (B) expression in patient samples Samples were obtained from patients with mild, moderate or severe dysplasia and invasive cervical carcinoma. LSIL – Low-grade squamous Intraepithelial Lesion, HSIL – High-grade squamous Intraepithelial Lesion (according to Bethesda system terminology). **p < 0.001; # *p* < 0.05.

Immunohistochemical study of 37 patients' samples showed low E-cadherin expression in high-grade intraepithelial lesion (HSIL) and invasive carcinoma Figure 6A. To our surprise, a significant decrease in the E-cadherin was also observed for low-grade squamous intraepithelial lesion (LSIL) in comparison to normal cervix (Figure 6A, Supplementary Table 2). Slug expression was higher in HSIL and invasive carcinoma in comparison to LSIL (Figure 6B; Supplementary Table 3).

### MET receptor downregulation influences E-cadherin and slug expression

**Figure 7:**
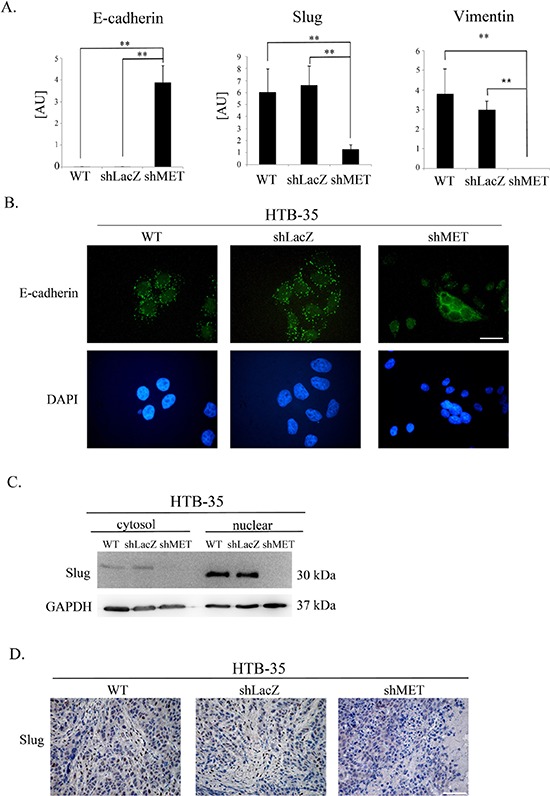
E-cadherin, SLUG and Vimentin expression after MET receptor downregulation Control cells had undetectable levels of E-cadherin (a hallmark of the epithelial phenotype) as well as high expression of SLUG (a driver of EMT) and Vimentin. MET receptor downregulation increased E-cadherin and decreased SLUG and Vimentin expression. **(A)** Real-time RT-PCR analysis of E-cadherin, SLUG and Vimentin expression. **(B)** Immunofluorescence analysis of E-cadherin levels and localization. **(C)** An analysis of SLUG expression revealed reduced levels of SLUG mRNA and undetectable SLUG protein in both the cytosolic and nuclear fractions after MET downregulation. **(D)** Immunohistochemical staining for SLUG revealed high expression in control cells, with primarily nuclear localization. Tumors formed by MET-deficient cells did not express SLUG. These experiments were performed at least three times. Representative staining is shown. Bar = 50 μm. **p < 0.001.

Because MET receptor downregulation in HTB-35 shMET cells led to a lower proliferation rate along with an altered cell shape and F-actin reorganization, we assumed that MET silencing at least partially reversed their phenotype into a less aggressive form. To expand on these observations, we evaluated potential changes in the expression of various EMT markers. MET receptor silencing upregulated E-cadherin and suppressed Slug and Vimentin mRNA expression in HTB-35 cells (Figure 7A). An immunofluorescence analysis of E-cadherin distribution showed that in control cells, E-cadherin was present in discrete, disrupted dots, whereas MET-deficient cells showed lateral membrane localization at sites of cell-cell contact (Figure 7B). A western blot analysis revealed that Slug protein levels were very low and absent in the cytosolic fractions of control and shMET HTB-35 cells, respectively. In the nuclear fraction, Slug expression was very strong in control cells but undetectable in MET-deficient cells (Figure 7C). Based on our *in vivo* observation that MET-deficient tumors were more differentiated than controls and re-expressed E-cadherin, we examined Slug expression in tumor specimens. This immunohistochemical analysis revealed that tumors formed by control HTB-35 cells presented high Slug expression in nuclei and low expression (pale staining) in the cytoplasm. By contrast, tumors derived from shMET HTB-35 cells exhibited no Slug expression in nuclei and very low expression in the cytoplasm (Figure 7D).

Our histopathological analysis showed that E-cadherin was highly expressed in tumors generated by both control and shMET HTB-34 cells (Figure 3B) and we were interested whether MET receptor downregulation affects expression of some EMT markers (Supplementary Figure 2). HTB-34 WT, shLacZ and shMET cells had similar, high level of E-cadherin at mRNA level. MET deficient HTB-34 cells showed decreased SLUG expression and undetectable level of Vimentin (Supplementary Figure 2A). Moreover, we observed that control and shMET HTB-34 cells have very low expression of CXCR4 (Supplementary Figure 2B).

### MET receptor downregulation impairs CXCR4 expression and activation

**Figure 8:**
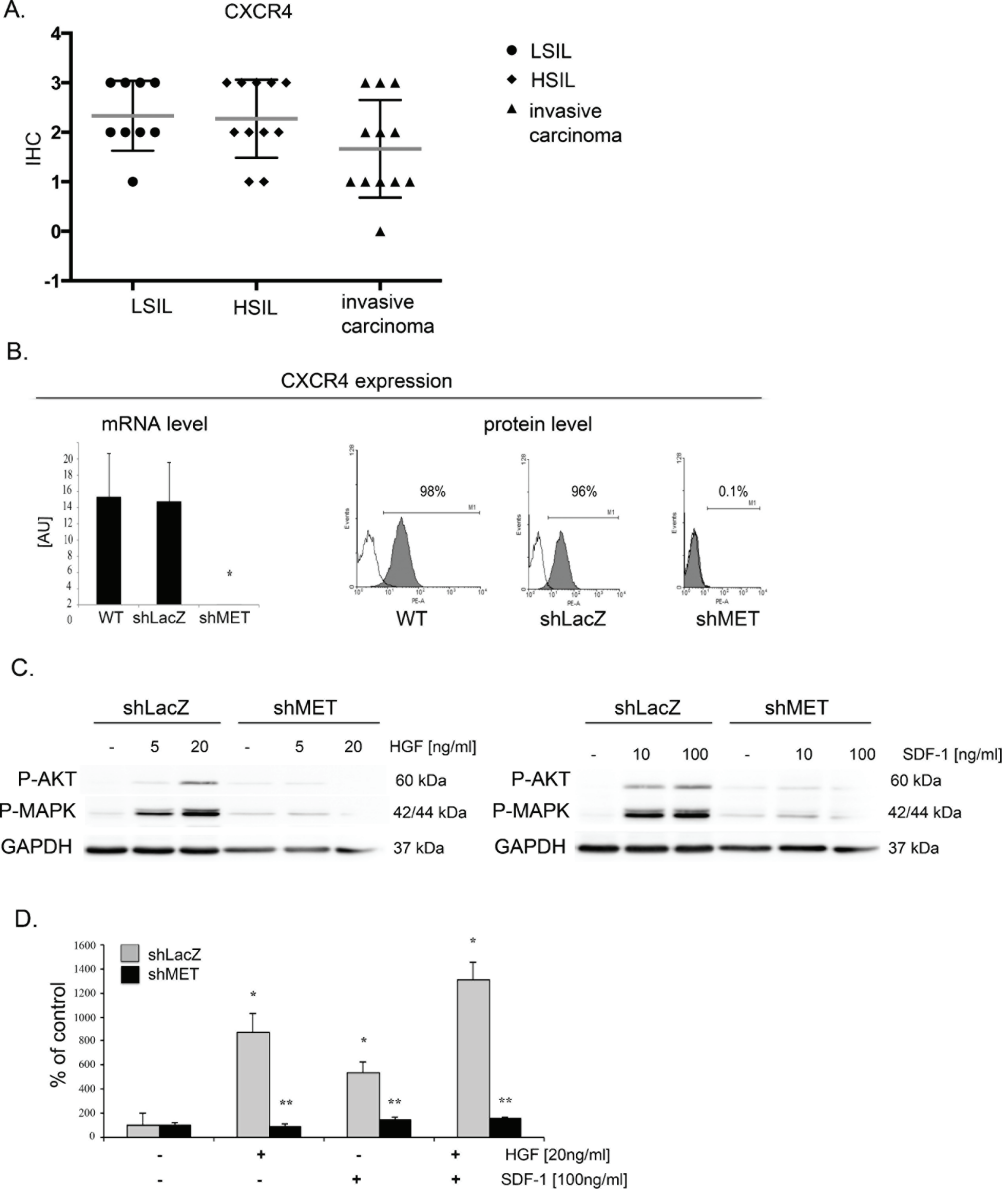
MET receptor downregulation decreases CXCR4 expression and function **(A)** Immunohistochemical analysis of CXCR4 receptor expression in human tissue. **(B)** Real-time RT-PCR and flow cytometry analysis revealed significant decreases in MET transcript and protein levels in MET receptor-silenced cells (shMET) relative to controls (wild type, WT, and shLacZ). **(C)** Western blot analysis of CXCR4 activation. AKT and MAPK phosphorylation was tested after after HGF and SDF-1 stimulation. **(D)** In chemotaxis assay MET-deficient CC cells showed limited chemotactic activity towards HGF and/or SDF-1 gradient. Western blot and FACS analyses were performed three times with similar results; representative results are shown. Real-time RT-PCR and chemotaxis assays were performed at least three times in duplicate. *p < 0.01, **p < 0.001.

SDF-1-CXCR4 axis is a key player responsible for invasive phenotype and metastatic behavior of many tumor types [30, 31]. In our study, we evaluated the level of CXCR4 expression in human samples (*n* = 31) obtained from cervical carcinoma patients. Regardless of the cancer stage, we observed strong and very strong expression of CXCR4 receptor (Figure 8A; Supplementary Table 4). We became interested whether downregulation of MET receptor influences SDF-1/CXCR4 axis. We noticed that the expression of CXCR4 was strongly decreased in shMET HTB-35 and unchanged in control HTB-35 cell lines (Figure 8B). After the activation of CXCR4 receptor with SDF-1 we found that control cells responded to the ligand by phosphorylation of AKT and MAPK kinases. However, the activation of MAPK and AKT after SDF-1 stimulation was highly impaired in shMET HTB-35 cells (Figure 8C). These data led us to the question how MET receptor influence the chemotactive activity of CXCR4 receptor. We found that downregulation of MET receptor drastically impaired directed migration towards HGF and SDF-1 ligands (Figure 8D). HTB-34 control and shMET cells did not express CXCR4 receptor (Supplementary Figure 2).

## DISCUSSION

Cervical cancer is the third most common tumor in women. According to WHO statistics, the worldwide mortality rate from cervical cancer is 52 percent [32, 33]. Global incidence and mortality rates depend upon the presence of human papillomavirus vaccination and screening programs for cervical cancer, which are more commonly available in developed countries. Developing countries thus carry the largest burden of cervical cancer, with more than eight out of ten (86%) cases diagnosed there in 2008 [34]. Therapy for advanced cervical cancer is still insufficient and presents a very important clinical problem.

In this paper, we examine the role of the MET receptor in the biology of cervical carcinoma. We show that MET receptor downregulation reduces the ability of tumor cells to grow *in vitro* and *in vivo* and causes a change in the cell phenotype to a more epithelial and less aggressive form, indicating the MET receptor as a potential therapeutic target.

Stimulation of the MET receptor via its natural ligand, HGF, results in scattering, angiogenesis, proliferation, enhanced cell motility, invasion and metastasis [35]. Overexpression of HGF and the MET receptor may also stimulate proliferation in cervical carcinoma cells [36]. Thus, we became interested in whether the MET receptor influences cell proliferation/viability under conditions of limited oxygen and nutrients that resemble the environment of a growing tumor. Our proliferation/viability analysis revealed that MET receptor downregulation significantly decreased the proliferation/viability of HTB-35 cervical carcinoma cells under these stress conditions. However, MET silencing did not noticeably alter cell proliferation/viability in two other cell lines. This discrepancy suggests that some cervical cancer patients will be more susceptible to MET blocking than others. Interestingly, based on our unpublished data, HTB-35 is the most aggressive of the three cell lines tested.

Our results further show that the constant presence of the MET receptor is crucial for maintaining a high level of proliferation in cervical cancer cells. Based on these findings, we became interested in whether the MET receptor could also influence the growth of cervical cells *in vivo* in NOD-SCID mice. We found that MET silencing in HTB-35 cells dramatically decreased tumor growth.

Our data thus suggest that HTB-35 cells depend on sustained MET activity for their growth and survival. This so-called “oncogene addiction” reveals a possible “Achilles' heel” of cancer cells, wherein they depend on a single oncogenic pathway or protein for sustained proliferation and/or survival [37, 38]. Dependence on MET has been previously shown by Comoglio's group, where MET could be genetically selected for the long-term maintenance of the primary transformed phenotype, and some tumors were dependent on sustained MET activity for their growth and survival [24, 25].

Our group has previously shown that MET downregulation causes a decrease in the size of tumors derived from rhabdomyosarcoma (RMS) cells transplanted into a NOD-SCID mouse model [23]. We also showed that MET receptor downregulation induces the differentiation of RMS tumors *in vivo* [39]. Other groups have also shown that MET silencing results in the impaired growth of other tumors, e.g., gastric and pancreatic carcinomas [40].

Here, we demonstrate for the first time that MET receptor downregulation inhibits cervical cancer cell growth *in vivo* in NOD-SCID mice. A histopathological evaluation revealed that these tumors were more differentiated than tumors derived from control cells. The MET receptor has been shown to be important for keeping cells of mesenchymal origin in an undifferentiated state (e.g., satellite cells) [41]. It is possible that during tumor development, some transdifferentiation takes place, which is more prominently revealed after MET downregulation. Our data suggest that the reduction in tumor size that occurs following MET receptor downregulation is, at least in part, caused by the differentiation of tumor cells. Our immunohistochemical analysis also revealed that MET receptor silencing induces the expression of E-cadherin in tumors formed by MET-deficient cells. To our knowledge, there have been no reports showing the re-expression of E-cadherin after MET receptor downregulation. E-cadherin is a transmembrane protein that is localized to adherens junctions along the basolateral surface and plays important roles in the maintenance of epithelial morphology and the repression of cell invasion and metastasis [42]. Loss of E-cadherin expression and/or function is a well-recognized marker of EMT. Previous studies showed that E-cadherin expression is significantly decreased in cervical carcinoma tissues and almost absent when tumor cells invaded deeply into the parametrium or spread to pelvic lymph nodes [43]. Our findings provide evidence that MET receptor downregulation in cervical cancer cells can lead to reversal of the EMT phenotype through re-expression of E-cadherin.

Our data also provide evidence that the MET receptor plays an important role in regulating the growth and invasive behavior of cervical carcinoma cells. These findings suggest that MET receptor expression or overexpression might contribute to the resistance of cervical cancer to conventional methods of treatment.

Tumor growth can be regulated by increased proliferation, increased survival or both. We show that MET downregulation decreased cervical cancer cell proliferation. In contrast to other studies where MET receptor silencing promoted apoptosis [44], we did not observe increased apoptosis in MET-deficient cervical carcinoma cells. Rather, we found that MET downregulation altered the cell-cycle distribution such that shMET cells accumulated in G2/M phase. This is likely a consequence of the decreased expression of c-myc and cyclin D1 observed in shMET cells. Interestingly, these results are consistent with our previous data where HGF upregulated cyclin D1 expression in cervical carcinoma cells [22].

When we examined the effect of MET downregulation on cell morphology and cytoskeletal organization, we observed a dramatic change in cell size in shMET HTB-35 cells. These cells were, on average, half the size of their control (WT and shLacZ) counterparts. Moreover, MET downregulation reorganized F-actin from thick parallel bundles into thin strands under the membrane. Actin filaments in epithelial cells are organized into thin cortical bundles, whereas they form thick contractile stress fibers in transdifferentiated mesenchymal cells [45]. This similarity suggests that MET-deficient HTB-35 cells have some epithelial characteristics.

To examine other mechanisms underlying the reduced tumor growth and change in cell morphology, we assessed the expression of EMT-related genes in MET-deficient cells. We found that MET receptor downregulation dramatically increased E-cadherin levels and redistributed this protein into the localization pattern characteristic of epithelial cells, where it is a key component of cell–cell adherens junctions [46]. The level of E-cadherin correlates with the expression level of the SLUG transcription factor. We observed that MET downregulation decreased Slug levels in cervical cancer cells both *in vitro* and *in vivo*, suggesting its role in regulating E-cadherin expression in cervical carcinoma cells.

Our results are consistent with previous reports showing that SLUG plays a major role in EMT during breast cancer metastasis through partial inhibition of E-cadherin [47]. Moreover, Bolos et al. found that stable expression of SLUG in MDCK cells led to the full repression of E-cadherin and triggered a complete epithelial-to-mesenchymal transition [48]. Interestingly, restoring E-cadherin expression in lung cancer cell lines increased sensitivity to the cytostatic drug, gefitinib [49].

The CXCR4/CXCL12 axis can coordinate metastasis of a variety of cancers [30, 31]. We have previously found that CXCR4 expression influenced invasive properties of cervical carcinoma cells both *in vitro* and *in vivo* [50].

In this study, we have also shown for the first time that blocking of MET receptor can influence expression and function of chemokine receptors. Cells with decreased MET receptor expression had downregulated expression of CXCR4 receptor. Moreover, these cells had impaired intracellular signaling and chemotaxis toward SDF-1 gradient, which is in accordance with decreased expression of CXCR4.

Until now, no published studies have evaluated the role of the MET receptor in promoting the EMT phenotype in cervical carcinoma. Our findings enable us to postulate that the MET receptor is a critical agent responsible for EMT and that blocking of MET receptor function could reverse this process and thus represent a new therapeutic strategy for advanced cervical cancer.

Our findings provide also evidence that MET receptor regulates growth and invasive behavior of cervical carcinoma cells. Thus, our data might suggest that MET receptor expression or overexpression could also contribute to the resistance of cervical cancer to conventional treatment.

## MATERIALS AND METHODS

### Cell lines

All cervical carcinoma cell lines used in this study: HeLa, HTB 34, HTB 35 were obtained from ATCC. Cells were maintained in MEM (Gibco BRL, Grand Island, New York, USA) supplemented with 10% heat-inactivated FBS (Gibco BRL, Grand Island, New York, USA), 100 IU/ml penicillin, 10 mg/ml streptomycin (Gibco BRL, Grand Island, New York, USA). Cells were cultured at 37^o^C, 5% CO_2_, 95% humidity. They were split usually twice a week with medium change.

### Analysis of human samples

Formalin-fixed, paraffin-embedded samples from patients with diagnosed cervical carcinoma or dysplasia of the cervix were evaluated histopatologically. The analysis included invasive SCCs (invasive squamous cell carcinoma) of the cervix (n-12), HSILs (high-grade squamous intraepithelial lesion) (n-11), LSILs (low-grade squamous intraepithelial lesion) (n-8) and normal cervix (n-9). Immunohistochemical evaluation was performed using primary antibodies: mouse monoclonal anti E-cadherin antibody, 1:25 Clone: NCH-38; M3612; (DakoCytomation, Denmark), mouse monoclonal anti CXCR4 (Clone 44716), 1:25 (R&D System, Minneapolis, MN, USA), rabbit monoclonal anti Slug, 1:25 (Cell Signaling, Danvers, Massachusetts, USA), rabbit polyclonal antibody anti MET, 1:50 (C-12: sc-10; Santa Cruz Biotech., Santa Cruz, California, USA) and EnVision™ Detection Systems Peroxidase/DAB, Rabbit/Mouse (DakoCytomation, Denmark).

The samples were analysed with the approval from the Bioethics Committee of the Jagiellonian University (no. KBET/7/B/2008).

In order to perform expression analysis of the investigated proteins we used the following scale: 0 (+/−) – very poor response/poorly positive discontinuous, 1 (+) – poor response, 2 (++) – moderate response, 3 (+++) – quite strong/strong response, 4 (++++) – very strong response.

### Lentiviral vectors construction, production and *in vitro* transduction

Lentiviral vectors production was performed as described earlier [23]. Shortly, the pENTR vector containing the MET- or LacZ-specific short hairpin RNA (shRNA) was recombinated with pLenti6/BLOCK-iT™-DEST expression vector (Invitrogen, Carlsbad, California, USA). MET shRNA sequence 5′–AGU CCG AGA UGA AUG UGA Att–3′ has been designed with use of available algorithm from Ambion http://www.ambion.com/techlib/misc/siRNA finder.html. High titer lentiviral vector stock was produced in 293FT cells by transient lipofection of the pLenti6-GW/U6-shRNA and packaging plasmids pLP1, pLP2, and pLP/VSVG. Tumor cells were transduced directly with viral supernatants and subsequently selected with blasticidin (Invitrogen, Carlsbad, California, USA). Human cervical carcinoma cells transduced with anti MET shRNA were indexed with shMET, transduced with anti LacZ – with shLacZ label.

### Analysis of cell morphology

In order to characterize the morphology of tumor cells, HTB-35 WT, shLacZ and shMET cells were seeded on cover slides and fixed with 2% of paraformaldehyde. Cells were photographed with Nomarski interference contrast optics and with epifluorescence mode. The area of cell surface projection, extension, elongation and dispersion were calculated as described by Dunn and Brown [51] with program MIGRA [version 1.1 RTO, by Ryszard Tokarski]. Extension is the measure of how much the shape differs from a circle. It takes *a* value of zero if the shape is circular and increases without limit as the shape becomes less compact [51]. Dispersion is the minimum extension that can be attained by compressing the shape uniformly. It takes *a* value of zero if the shape is circular [51]. Elongation is the measure of how much the shape must be compressed along its long axis to minimize its extension. It takes *a* value of zero if the shape is circular and increases as the shape becomes less compact [51]. The mean value was obtained from analysis of 50 cells. Statistically significant at ## *p* < 0.005.

### Cell proliferation

Cell proliferation was measured by using the [3-(4,5-dimethylthiazol-2-yl)-2,5-diphenyl] tetrazolium bromide (MTT) assay and cell counting. MTT assay was performed according to the manufacturer's recommendations (Promega, Madison, Wisconsin, USA). Briefly, the cells were seeded in triplicate or sextuples in flat-bottomed 96-well plates at 1 000 cells per well and allowed to adhere for 24 hours in serum supplemented (10%) media. Thereafter, cells were cultured under hypoxia (2% O_2_, 10% FBS) and starvation (0.5% BSA) conditions. After 24, 48, 72 and 96 hours, 20 μl of CellTiter 96 Aqueous One Solution reagent were added to each well and plates were incubated for 4 hours. Subsequently, plates were read at 490 nm using the ELx800 Universal Microplate Reader (Bio-tech) and analyzed with KC4 v3.0 with PowerReports software. For cell counting, cells were seeded in 24-well plates at 2 × 10^4^/well. After 24, 48, 72 and 96 hours, cell were dissociated with 0.5% trypsin/EDTA and counted in a hemocytometer. Each experiment was repeated trice and each concentration was in triplicates. **p < 0.001 was considered as significant.

### RNA extraction and reverse transcription

Total RNA was extracted using RNeasy Mini Kit (Qiagen, Valencia, California, USA) followed by DNAse treatment (Promega, Madison, Wisconsin, USA). The reverse polymerase transcription was performed using MMLV reverse transcriptase (Invitrogen, Carlsbad, California, USA) according to manufacturer's protocol.

### Quantitative real time PCR analysis

Determination of complementary DNA (cDNA) levels was performed by real-time polymerase chain reaction on an ABI PRISM 7300 Sequence Detection System (Applied Biosystems, Foster City, CA, USA). Specific primers probe sets were purchased from Applied Biosystems (Foster City, CA, USA). cDNA expression level for all samples was normalized to the housekeeping gene glyceraldehyde phosphate dehydrogenase (GAPDH). The probes sequences were as follows: Human: GAPDH (Hs99999905_m1), MET (Hs01565589_m1), c-myc (Hs00153408_m1), cyclin D1 (Hs00765553_m1), Slug (Hs00950344), E-cadherin (Hs01023894m1), CXCR4 (Hs00237052_m1). 2^−ΔΔCT^ method allowed to calculate relative expression of the genes.

### Chemotaxis assay

The directional movement of cells toward HGF and SDF-1 gradient was evaluated using modified Boyden's chamber with 8 μm pore polycarbonate membrane inserts (Transwell; Costar-Corning, Cambrige, MA, USA). Cells, detached with 0.25% trypsin, were seeded into the upper chamber of an insert at a density of 2.5 × 10^4^ in 100 μl, in duplicates. The lower chamber was filled with pre-warmed medium containing HGF (20 ng/ml) (R&D System, Minneapolis, MN, USA) or SDF-1 (10, 100 ng/ml) (PeproTech EC, London, UK). 0.5% BSA DMEM medium was used as a negative control. After 24 hours, inserts were removed from the transwell and cells were fixed with methanol. Cells that did not migrate were scraped off with cotton wool from the upper membrane and cells that had transmigrated to the lower side of the membrane were stained with Wright solution and counted under high power field (HPF) with inverted microscope. At least five fields were counted each time and the mean number of cells per HPF was calculated. Chemotaxis assay was performed at least three times, always in duplicates.

### Flow cytometry

Cells were recovered from plates with Accutase (PAA Laboratories, Linz, A) and stained with antibody against MET (monoclonal FITC-labeled anti-human HGFR/c-MET antibody, clone 95106 (R&D System, Minneapolis, MN, USA) or monoclonal PE-labeled anti-human CD184 (CXCR4) antibody, clone 12G5 (BD Pharmingen, San Diego, CA, USA). Briefly, 1 × 10^5^ cells suspended in 100 μL staining buffer (PBS, 2% FBS) were added to a test tube containing the appropriate amount of each antibody. Cells were incubated in the dark for 30 minutes at 4°C. Stained cells were washed and collected using a FACSCanto cytometer (Becton Dickinson) and analyzed with FACS Diva software (BD Pharmingen, San Diego, CA).

### Cell cycle analysis

Cells were seeded in 100 mm dishes at a density of 5 × 10^5^ per dish. Twenty-four hours later, medium was changed and after 48 hours both adherent and floating cells were harvested and lysed for 10 minutes in lysing buffer (0.1% sodium citrate) (Sigma, St Louis, Missouri, USA), 0.1% Triton X-100 (Sigma, St Louis, Missouri, USA), 40 μg/ml Propidium Iodide (Sigma, St Louis, Missouri, USA). Cells were analyzed for DNA content by flow cytometry with FACSCanto Cytofluorometer (BD Pharmingen, San Diego, CA) and the cell-cycle phases were analyzed using FCS Express V3 with MultiCycle AV (http://www.denovosoftware.com). The experiments were performed twice with similar results.

### Apoptosis assay by flow cytometry

Apoptosis was measured by flow cytometry using an Annexin V-FITC Apoptosis Detection kit I (BD Pharmingen, San Diego, CA). Briefly, cells were cultured in MEM supplemented with 10% FBS, 1 × 10^6^ cells were washed with cold PBS and then resuspended in 1 × cold binding buffer plus 5 μl Annexin V-FITC and 5 μl PI (propidium iodide). After 15 min incubation at RT in the dark, samples were analyzed by dual color flow cytometry on a FACSCanto Cytofluorometer (BD Pharmingen, San Diego, CA).

### Western blot

Western blots were done on extracts prepared from cells as described previously [39, 50]. Briefly, CC cells were lysed (for 10 min) on ice in M-Per lysing buffer (Pierce, Rockford, Illinois, USA) containing protease and phosphatase inhibitors (Sigma, St Louis, Missouri, USA). Subsequently, the extracted proteins were separated on a 12% sodium dodecyl sulfate-polyacrylamide gel electrophoresis (SDS-PAGE) gel, and fractionated proteins were transferred into a PVDF membrane (BioRad; BioRad Laboratories, Hecules, CA, USA). Total level of c-myc was detected by primary rabbit polyclonal antibody (Cell Signaling, Danvers, Massachusetts, USA). Total level of cyclin D1 and Slug was assessed using rabbit monoclonal antibodies (Cell Signaling, Danvers, Massachusetts, USA). The level of Slug was detected using rabbit monoclonal anti-Slug antibody, (Cell Signaling, Danvers, Massachusetts, USA). The phosphorylation of AKT and MAPK was assessed using primary rabbit anti-phospho-AKT (Ser 473) and mouse anti-phospho-MAPK (Thr202/Tyr204) antibodies both from Cell Signaling Danvers, Massachusetts, USA. They were subsequently detected with horseradish peroxidase (HRP)-conjugated goat anti-rabbit or anti-mouse secondary antibody (Santa Cruz Biotech., Santa Cruz, California, USA). The membranes were developed with an enhanced chemiluminescence (ECL) reagent (Amersham Life Sciences, Little Chalfont, UK), dried, and subsequently exposed to the HyperFilm (Amersham Life Sciences, Little Chalfont, UK). An equal loading in the lanes was evaluated by probing with rabbit anti-GAPDH from Cell Signaling, Danvers, Massachusetts, USA.

### Fluorescence microscopy studies

For fluorescence microscopy studies, cells were plated on glass coverslips. 48 hours after seeding cells were fixed with 4% paraformaldehyde in PBS for 30 min at room temperature (RT). For F-actin staining, cells were incubated with Alexa 488–conjugated phalloidin (Sigma, St Louis, Missouri, USA), (1:500) diluted in 0.1% BSA for 1 h. To detect E-cadherin cells were permeabilized in PBS containing 1% triton X-100 for 20 min, blocked with 1% BSA in PBS for 30 min and then incubated with primary anti-E-cadherin antibody (BD Pharmingen, San Diego, CA, USA) (1:50) diluted in 1% BSA, overnight at 4^°^C. After several washes with PBS, the samples were incubated with Alexa Fluor 488-conjugated anti-mouse secondary antibody (1:1000) (Invitrogen, Carlsbad, California, USA) for 1 h at RT and embedded in Vectahield (Vector Laboratories, Burlingame, CA, USA). Cells were visualized in an Olympus BX-51 microscope with the appropriate filters. Representative images were taken with a Spot 4.3 digital camera and software and edited in Adobe Photoshop.

### Long-term murine models

For long term-assay 5 × 10^6^ CC cells were injected subcutaneously into 6–8 weeks NOD-SCID mice. After 30 days mice were killed and their tumors were harvested. Tumors were weighted and fixed in formalin. Immunohistochemical evaluation was performed using anti-E-cadherin monoclonal primary antibody (Clone: NCH-38; M3612; DakoCytomation, Denmark) and anti-Slug rabbit monoclonal antibody (Cell Signaling, Danvers, Massachusetts, USA) and LSAB+ Kit (DakoCytomation, Carpinteria, CA, USA). Each experimental group comprised of 5 animals and experiments were repeated at least three times. **p < 0.001 was considered as significant. All experimental procedures were carried out according to the Jagiellonian University guidelines for the use and care of laboratory animals and were approved by I Local Ethics Committee in Krakow (no. 55/2011).

### Statistical analysis

Statistical analysis, unless otherwise indicated, was performed using the two-tailed Student's *t*-test. All experiments were performed at least three times (chemotaxis analysis and real-time RT-PCR additionally in duplicates). *p* values, lower than 0.01 (*), 0.001 (**), 0.05 (#), 0.005 (##), were considered as significant.

## SUPPLEMENTARY FIGURES AND TABLES


