# Supplementary material for: MET receptor is a potential therapeutic target in high grade cervical cancer
Source: Oncotarget. 2015 Apr 4;6(12):10086–101. doi: 10.18632/oncotarget.3161 (PMC4496342; doi:10.18632/oncotarget.3161)
Supplement: Supplementary file 1 [file oncotarget-06-10086-s001.pdf]

SUPPLEMENTARY FIGURES AND TABLES

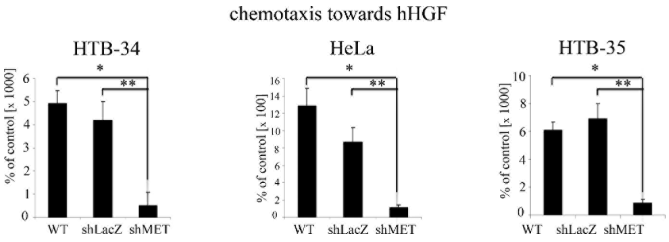

Supplementary Figure 1: The influence of MET receptor downregulation on chemotactic response of cervical cancer cells. MET receptor downregulation via a lentiviral vector containing anti-MET shRNA resulted in a change on functional levels. Chemotaxis assay of CC cell lines. MET-deficient CC cells show limited chemotactic activity towards an HGF gradient after stimulation with 20 ng/ml of HGF. Chemotaxis assays was performed at least three times in duplicate. *$p < 0.01$, **$p < 0.001$.

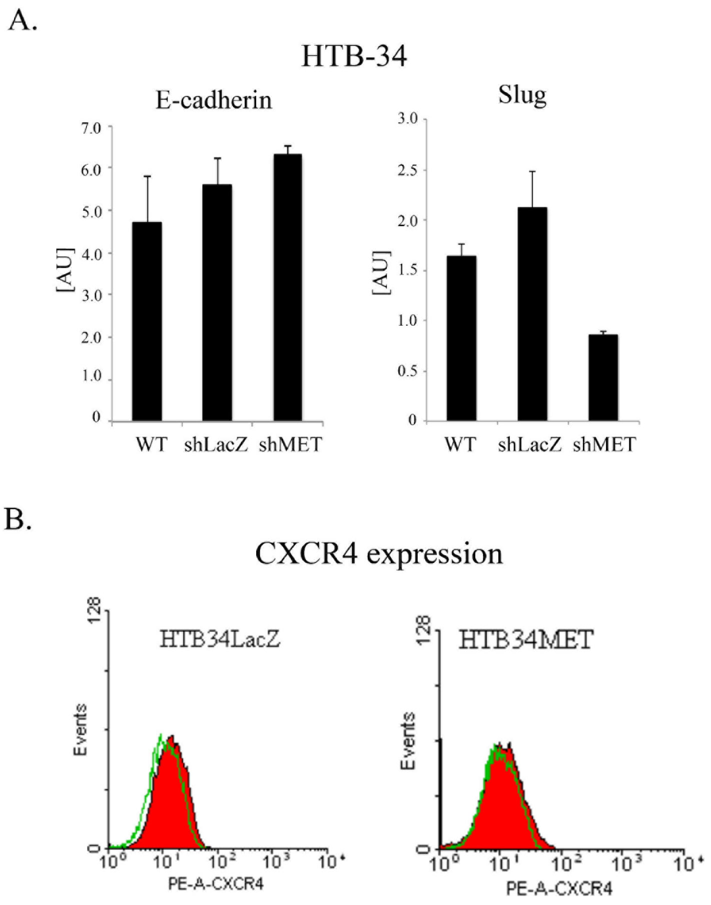

Supplementary Figure 2: E-cadherin, SLUG and CXCR4 expression after MET receptor downregulation. HTB-34 WT, shLacZ and shMET had similar, high level of E-cadherin. MET receptor downregulation decreased SLUG expression. HTB-34 control and shMET cells had undetectable level of Vimentin (data not shown) as well as very low expression of CXCR4 receptor. **(A)** Real-time RT-PCR analysis of E-cadherin and SLUG expression. **(B)** Flow cytometry analysis of CXCR4 expression. Real-time RT-PCR experiments were performed three times in duplicates. Flow cytometry analysis was performed three times and representative results are shown.

Supplementary Table 1: The influence of MET receptor downregulation on gene expression

GENE	Experiment 1			Experiment 2		
	WT	shLacZ	shMET	WT	shLacZ	shMET
**HGF**	0,0005	0,0003	**0,0030**	0,0020	0,0006	**0,0050**
**c-myc**	29,2788	8,4072	**8,7621**	146,7213	150,3191	**32,6460**
**CXCR4**	6,7420	6,6161	**0,0002**	16,0860	17,1173	**0,0000**
**CXCR7**	0,0018	0,0017	**6,9463**	0,0041	0,0027	**7,3187**
**Snail**	3,3841	2,5652	5,6210	2,6063	2,3823	6,3268
**Slug**	5,9042	5,4954	**1,2136**	9,2701	8,7987	**1,7297**
**Twist**	0,0000	0,0001	**0,0012**	0,0000	0,0002	**0,0033**
**E-cadherin**	0,0073	0,0071	**3,7674**	0,0193	0,0065	**3,2504**
**VEGF-A**	6,9323	7,2342	7,9430	18,3148	13,3719	13,2070
**IL-8**	0,2406	0,0768	0,0362	0,2005	0,0662	0,2709
**HIF-1α**	35,3254	45,0822	**217,4944**	44,6128	34,9237	**252,8581**
**MMP-2**	0,0017	0,0007	**0,0265**	0,0021	0,0012	**0,0678**
**MMP-9**	0,0005	0,0000	**0,0014**	0,0008	0,0006	**0,0027**
**TIMP-1**	37,8399	22,1792	**100,5371**	46,5782	26,8814	**128,6791**
**TIMP-2**	19,4330	20,1776	**89,0957**	16,3825	14,9061	**101,6794**

Real-time RT-PCR analysis of gene expression in control (WT and shLacZ) and MET – depleted cells. mRNA relative expression level [AU].

Supplementary Table 2: Immunohistochemical analysis of E-cadherin expression in human samples

E-cadherin	+/–	+	++	+++	++++
**Normal cervix**	0%	0%	22.2%	77.8%	0%
**LSIL**	0%	66.7%	33.3%	0%	0%
**HSIL**	33.3%	55.6%	11.1%	0%	0%
**Invasive carcinoma**	60%	40%	0%	0%	0%

In order to perform expression analysis of E-cadherin we used the following scale: 0 (+/–) - very poorly positive/ poorly positive discontinuous, 1 (+) – poor response, 2 (++) – moderate response, 3 (+++) – quite strong/strong response 4 (++++) – very strong response; *n* = 37.

Supplementary Table 3 : Immunohistochemical analysis of Slug expression in human samples

Slug	+/–	+	++	+++	++++
**LSIL**	0%	8.3%	66.7%	22.2%	0%
**HSIL**	0%	0%	30%	70%	0%
**Invasive carcinoma**	0%	8.3%	16.7%	75%%	0%

In order to perform expression analysis of Slug protein we used the following scale: 0 (+/–) - very poorly positive/ poorly positive discontinuous, 1 (+) – poor response, 2 (++) – moderate response, 3 (+++) – quite strong/strong response 4 (++++) – very strong response; *n* = 31

Supplementary Table 4: Immunohistochemical analysis of CXCR4 receptor expression in human samples

CXCR4	+/–	+	++	+++	++++
**LSIL**	0%	11%	44.5%	44.5%	0%
**HSIL**	0%	18.2%	36.4%	45.4%	0%
**Invasive carcinoma**	8.3%	41.7%	25%	25%%	0%

In order to perform expression analysis of CXCR4 protein we used the following scale: 0 (+/−) – very poorly positive/poorly positive discontinuous, 1 (+) – poor response, 2 (++) – moderate response, 3 (+++) – quite strong/strong response 4 (++++) – very strong response; $n = 31$.
